# Book Reviews

**Published:** 1800-03

**Authors:** 


### MISCELLANEOUS.

*An Eſſay on the Preſervation of Shipwrecked Mariners, in Anſwer to the Prize Queſtions propoſed by the Royal Humane Society, &c.* By A. Fothergill, M. D. F. R. S. &c. 8vo. pp. 61. 2s. 6d. London. Nichols.

The importance of the ſubject, particularly to this country, will, undoubtedly, procure this ſmall work a very extenſive circulation; and as a ſpecial meeting of the Directors of the Royal Humane Society have reſolved unanimouſly, " That it is a very able and ſcientific performance, happily enliſting Philoſophy in the cauſe of Humanity,"—the following extracts appear to us to contain uſeful hints:

" The treatment of ſhip-wrecked mariners, when nearly exhauſted by cold and hunger, demands no ſmall circumſpection. At firſt, they loath the ſight of meat; but on a ſudden the appetite grows voracious, and prompts them to devour more food than the digeſtive organs can aſſimilate. Their native warmth alſo, being greatly impaired, ſhould be very gradually reſtored by a tepid bath; and the food ſhould conſiſt of new milk, barley water, or weak broth, in very ſmall quantity, which, in this ſtate of more than infantile debility, will be found ſufficiently powerful; avoiding, at the ſame time, the common error of pouring down wine, ſpirits, or other ſtimulating cordials, which, inſtead of ſupporting life, too often exhauſt the feeble remains of vitality."

The account given of the *life-boat* at Shields, and its ſingular utility, evinced by repeated trials, is ſo intereſting, that we ſhall extract from it what relates to its practical uſe. Mr. Fairleſs has furniſhed furnished some useful hints respecting the original plan, and describes this vessel, as follows:

"It measures 30 feet by 10, resembles in its form a common Greenland boat, only flatter in the bottom. The weight of cork employed in the construction is about 7 cwt. with which the boat is lined, inside and outside of the gunwales, two feet in breadth; the seats being also filled with the same: rowed by ten men, double banked, and steered by one at each end with oars, being alike at both ends, and with a contrivance to prevent sinking in the sand.

"She draws very little water, and can carry twenty persons, even when full of water. Being water proof, and rendered buoyant by cork, she keeps afloat, preserving her equilibrium without danger of oversetting, and is able to contend against the most tremendous sea, having never, in any one instance, yet failed of conveying a distressed ship's crew into safety.

"In going off with her, in the highest seas and broken water, the men testify no dread; and though cork-jackets were provided for them, yet such is their confidence in the boat, that they now refuse to use them.

"Indeed she has surprized every intelligent seaman that has seen her contend with the boisterous waves. Any farther description I can give," adds Mr. Fairless, "will not be equal to a view of the model kept at Northumberland House, in London, which, I make no doubt, you may see. Such a vessel ought to be provided with high wheels, to convey it to the place where it is immediately wanted.

"The boat complete, and copper nailed, costs about 150l. But, in the moment of distress, what shipwrecked mariner would not think this a cheap purchase, and pronounce the value of such a boat as truly inestimable!"

---

*The Anatomist's Vade-mecum; containing the Anatomy and Physiology of the Human Body. The second edition, revised and enlarged,* by R. Hooper, M. D. F. L. S. &c. 12mo. pp. 219. 3s. 6d. London, Murray and Highley.

The learned and accurate author of this work explains his views in a short introduction prefixed to it. "It is the intention of the writer, in the following compendium, to present to the student an useful Anatomical Conspectus, or Pocket Manual of Anatomy and Physiology; giving a short but accurate description of the different parts of the human body and their functions; with a glossary, or explanation of the principal terms used in that science."

The utility of this performance, in the English language, as well as the manner in which it is executed, induce us to recommend it to all students of Anatomy and Surgery.

---

*The*

*The Villager's Friend and Phyſician; or, a familiar Addreſs on the Preſervation of Health, and the Removal of Diſeaſe on its firſt Appearance*; by JAMES PARKINSON, 12mo. pp. 85, price 1s. London, Symonds.

The benevolent intention of the Author muſt be obvious to every Reader capable of appreciating the merit of this ſhort Addreſs; even the paper on which it is printed, ſeems calculated to facilitate its circulation: but we fear that not one in a thouſand of thoſe for whoſe benefit it is written, will ever obtain a ſight of it.

---

*An Eſsay to elucidate the Nature, Origin, and Connexion of Scrophula and glandular Conſumption; including a brief Hiſtory of the Effects of Ilkley Spa; with Obſervations on the medicinal Powers of Digitalis*; by G. MOSSMAN, M. D. 8vo. pp. 107. London, Johnſon, &c.

We recommend this Eſſay to our Readers, not ſo much on account of the novelty of opinion advanced in it, as the diligence with which the preſent opinions are collected and exhibited.

